# Explore the impact of hypoxia-related genes (HRGs) in Cutaneous melanoma

**DOI:** 10.1186/s12920-023-01587-8

**Published:** 2023-07-08

**Authors:** Guolin Ke, Nan Cheng, Huiya Sun, Xiumei Meng, Lei Xu

**Affiliations:** 1 Department of Dermatology and Venereology, Yijishan Hospital, Wannan Medical College, No. 2 Zheshan West Road, Wuhu City, Anhui Province, China; 2 Department of Hand, Foot, and Ankle Surgery, Yijishan Hospital, Wannan Medical College, No. 2 Zheshan West Road, Wuhu City, Anhui Province, China

**Keywords:** Cutaneous melanoma (CM), Hypoxia, ICIs

## Abstract

**Background:**

Cutaneous melanoma (CM) has an overall poor prognosis due to a high rate of metastasis. This study aimed to explore the role of hypoxia-related genes (HRGs) in CM.

**Methods:**

We first used on-negative matrix factorization consensus clustering (NMF) to cluster CM samples and preliminarily analyzed the relationship of HRGs to CM prognosis and immune cell infiltration. Subsequently, we identified prognostic-related hub genes by univariate COX regression analysis and the least absolute shrinkage and selection operator (LASSO) and constructed a prognostic model. Finally, we calculated a risk score for patients with CM and investigated the relationship between the risk score and potential surrogate markers of response to immune checkpoint inhibitors (ICIs), such as TMB, IPS values, and TIDE scores.

**Results:**

Through NMF clustering, we identified high expression of HRGs as a risk factor for the prognosis of CM patients, and at the same time, increased expression of HRGs also indicated a poorer immune microenvironment. Subsequently, we identified eight gene signatures (FBP1, NDRG1, GPI, IER3, B4GALNT2, BGN, PKP1, and EDN2) by LASSO regression analysis and constructed a prognostic model.

**Conclusion:**

Our study identifies the prognostic significance of hypoxia-related genes in melanoma and shows a novel eight-gene signature to predict the potential efficacy of ICIs.

**Supplementary Information:**

The online version contains supplementary material available at 10.1186/s12920-023-01587-8.

## Background

As a highly malignant skin tumor, the incidence of melanoma (CM) is increasing yearly, mainly due to the high metastasis rate, resulting in poor prognosis of patients [1]. Although early CM can be cured by local excision, metastatic CM is still a fundamental reason for threatening the health of patients [2]. Immune checkpoint inhibitors (ICIs), such as nivolumab, can act against programmed cell death-1 (PD-1) antibodies to treat many malignancies, including CM [3, 4]. However, there are still some patients who do not achieve good results. Therefore, identifying new biomarkers that can predict treatment response is particularly important for the rational use of ICIs.

The tumor microenvironment (TME) is also an essential part of tumor progression study, including metabolism, immune cell infiltration, biosynthesis, and physicochemical environment [5]. TME and tumor cells interact partly from diffusible metabolites [6]. Due to faster metabolism and proliferation in tumor tissue, increased aerobic glycolysis is required to obtain more energy, also known as the "Warburg effect" [7, 8]. Hypoxia is one of the basic features of TME due to the markedly increased oxygen consumption of tumor tissue. Studies have shown that hypoxia can cause heterogeneous changes, genetic instability, angiogenesis, and resistance to therapy, which can significantly affect the prognosis of cancer patients [9] and substantial tumors [10]. In CM, the relationship between hypoxia-related genes (HRGs) and immune cell infiltration, especially the potential efficacy of ICIs, has not been reported in detail.

In the present study, we used the Non-negative matrix factorization consensus clustering approach to investigate the relationship between HRGs prognosis and immune cell infiltration in CM patients. Subsequently, we finally identified eight gene signatures to construct a prognostic model through univariate COX analysis combined with the least absolute shrinkage and selection operator (LASSO) analysis. Finally, we performed a risk score for CM patients. We explored the relationship between the risk score and the potential surrogate markers of ICIs (such as TMB, IPS values, and TIDE scores) in CM patients, providing new insights into CM's assessment, treatment, and prognosis.

## Methods

### Data download

Complete gene expression data, clinical information, and mutation data, including 471 melanoma samples, were downloaded from the TCGA online database (https://portal.gdc.cancer.gov/). The GSE65904 dataset was published in April 2015 and was based on the GPL10558 platform. Resected tumors from 214 unique melanoma samples were profiled on gene expression arrays. The characteristics of the patients were presented in Supplementary Table 1. Patients without complete survival data were eliminated. Finally, 210 patients were included in this study. The tmb function of the R software package “maftools” was used to calculate each tumor mutation burden (TMB). In addition, we collected HRGs from the MSigDB website (https://www.gsea-msigdb.org/gsea/msigdb/).

### Non-negative matrix factorization consensus clustering

To investigate the role of HRGs in melanoma, we clustered melanoma samples from TCGA into two distinct clusters (cluster 1 and cluster 2) using NMF. NMF aims to identify latent features in gene expression profiles by parsing the original matrix into two non-negative matrices [11]. The depositions were performed iteratively, and their results aggregated to obtain consensus clusters of PTC samples. Determine the most appropriate number of subtypes based on the co-benzene coefficient, dispersion coefficient, and silhouette coefficient. NMF is performed by an R package named “NMF” [12]. We also used an R package called "survival" to compare the OS and DFS between clusters 1 and 2.

### Analysis of tumor immune microenvironment infiltration

In R, melanoma samples were analyzed using the "MCPcounter" package to obtain an overall score for immune cells. This method allows robust quantification of the absolute abundance of eight immune cell populations and two stromal cell populations in heterogeneous tissues based on transcriptomic data [13]. Subsequently, according to different clusters, the immune cell infiltration levels were compared, and violin plots were drawn using the "ggpubr" package.

### Prognostic model construction

We first used univariate COX regression analysis to identify the prognostic status of HRGs in melanoma. We randomly divided the melanoma samples into two groups (train group and test group), accounting for 70% and 30%. Preliminarily screened prognosis‐related HRGs were further narrowed down using the least absolute shrinkage and selection operator (LASSO) analysis through the “glmnet” R package. According to the prognostic model, the prognostic risk score of each train group and test group samples is calculated, and the samples are divided into a low-risk group and a high-risk group according to the median risk score.

### Survival analysis

Using the "survival" and "survminer" packages, plot survival curves for samples from the high and low-risk groups in the total sample group, the train group, and the test group. Subsequently, the receiver operating characteristic (ROC) curve was carried out using the R package “ROC.”

### Gene Set Enrichment Analysis (GSEA)

The transcriptome data and risk groups information was performed in R software GSEA (10.1073/pnas.0506580102). We selected the hallmark, c2.cp.kegg.v7.4.symbols.gmt in the MSigDB database as the reference gene set and screened out the top 5 enriched pathways in the high-risk group and the low-risk group for display.

### Relationship between HRGs and melanoma immune response

The Estimation of Stromal and Immune Cells in Malignant Tumors using the Expression Data (ESTIMATE) algorithm takes advantage of the unique properties of the transcriptional profiles to infer the tumor cellularity as the tumor purity. The relationship with Risk Score and StromalScore, ImmuneScore and ESTIMATEScore is plotted as a violin diagram in R using the "violin" package. The Cancer Immunome Atlas database (TCIA), which can be found at https://tcia.at/, is a comprehensive database that includes tumors from 20 different solid cancers. The database employs gene enrichment analysis and deconvolution techniques to calculate the composition of individual immune cells. Additionally, users can access information on the gene expression of specific immune-related gene sets, the composition of tumor-infiltrating immune cells (TIICs), new tumor antigens and cancer lineage antigens, HLA types, and tumor heterogeneity. The database is designed to provide insight into the features of infiltrating immune cells that respond to tumor genotypes, which determine immunophenoscores (IPSs) and immune escape mechanisms [14]. IPS-PD1/PD-L1 blocker and IPS-CTLA4 blocker data on CM from TCGA were obtained in TCIA for predicting patients’ potential surrogate markers of ICIs in high- and low-risk groups. Meanwhile, Tumor Immune Dysfunction and Exclusion (TIDE) algorithm predicted ICB response and evaluates immune escape ability. The TIDE scoring system is an essential method for predicting patient responses to anti-PD-1/L1 and anti-CTLA4 treatments by estimating T cell dysfunction and tumor immune evasion [15]. To calculate the TIDE score of each CM patient, we used an online tool available at http://tide.dfci.harvard.edu/. Based on the TIDE scores, we determined the differences between the high- and low-risk groups. We further analyzed TMB correlations for different risk grced them as boxplots using "ggplot2".

### Statistical analysis

R software (version 4.1.3, https://www.r-project.org/) and associated R packages were used to perform all graphical and statistical analyses. The Wilcoxon rank-sum test presents comparisons between the two groups, while the Kruskal–Wallis test assesses multiple comparisons. Multivariate cox regression analysis is used to evaluate the association between OS and clinicopathological characteristics and risk scores. Survival analysis was performed using the log-rank test. *P* < 0.05 was considered statistically significant.

## Result

### Non-negative matrix factorization consensus clustering

**Fig. 1:**
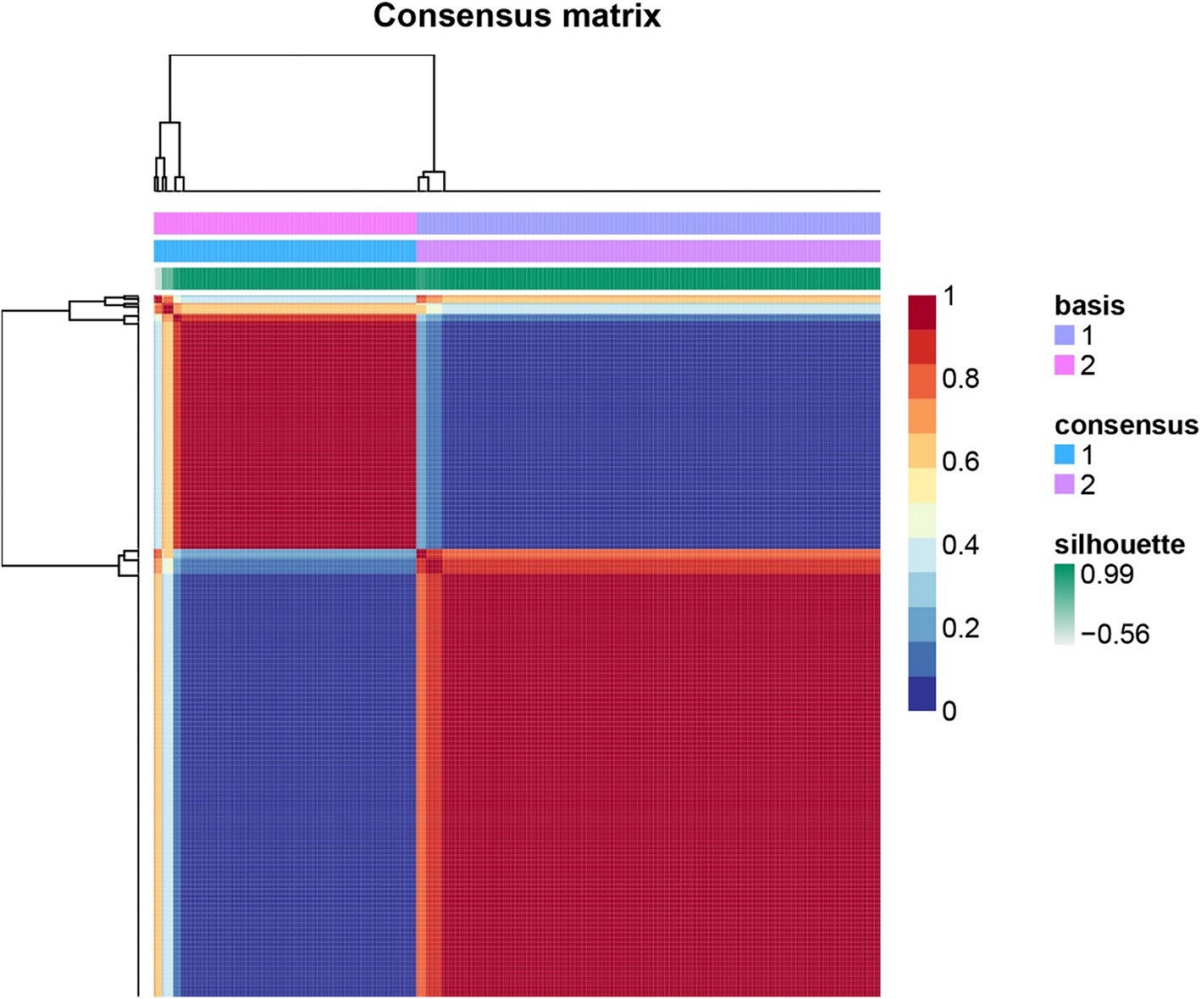
NMF clustering heatmap

To discover the role of HRGs in melanoma, we first performed a preliminary classification of molecular subgroups using NMF consensus clustering (Supplementary Figs. 1 and 2) based on 200 HRGs obtained from MSigDB. The entire TCGA cohort was clustered into two subgroups (cluster 1: *n* = 290 and cluster 2: *n* = 164), because when k = 2, the consensus matrix heat map maintained a clear and sharp boundary, indicating that the samples had stable and robust clusters (Fig. 1). Similarly, the heat map of the expression of HRGs in different clusters shows that there are large differences in the two clusters (Supplementary Fig. 3).

### Survival analysis

**Fig. 2:**
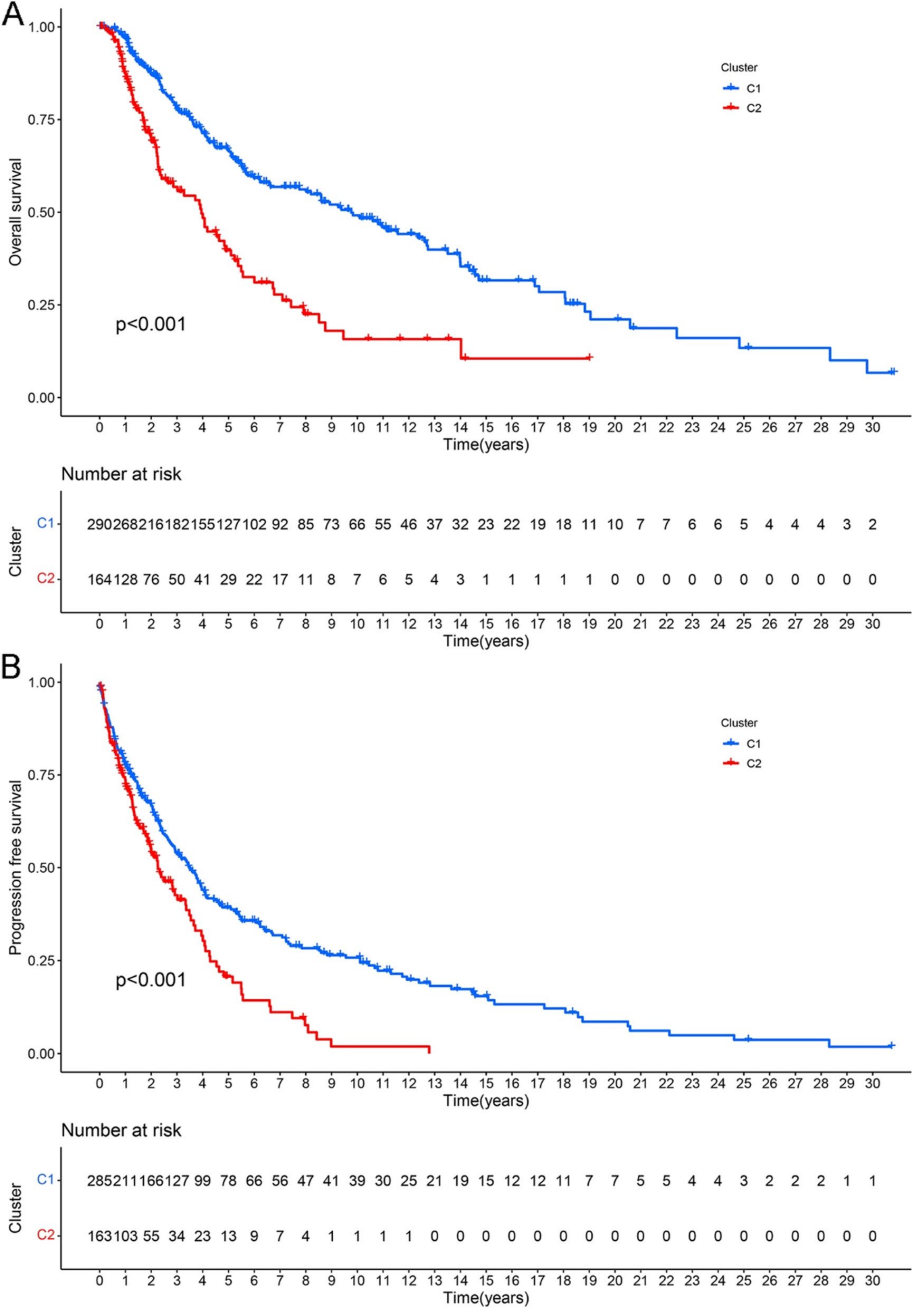
Survival curves for two groups of clustered samples. **A** Overall survival curve of CM patients. **B** The curve of disease-free survival in CM patients

To better understand the clustering results and their relationship to survival outcomes and clinical phenotypes, we compared OS and DFS between clusters 1 and 2. We found that cluster 1 had better OS and DFS than cluster 2 (*P* < 0.001, Fig. 2A, B). This result indicates that HRGs strongly correlate with the prognosis of melanoma patients.

### HRGs are associated with immune cell infiltration.

**Fig. 3:**
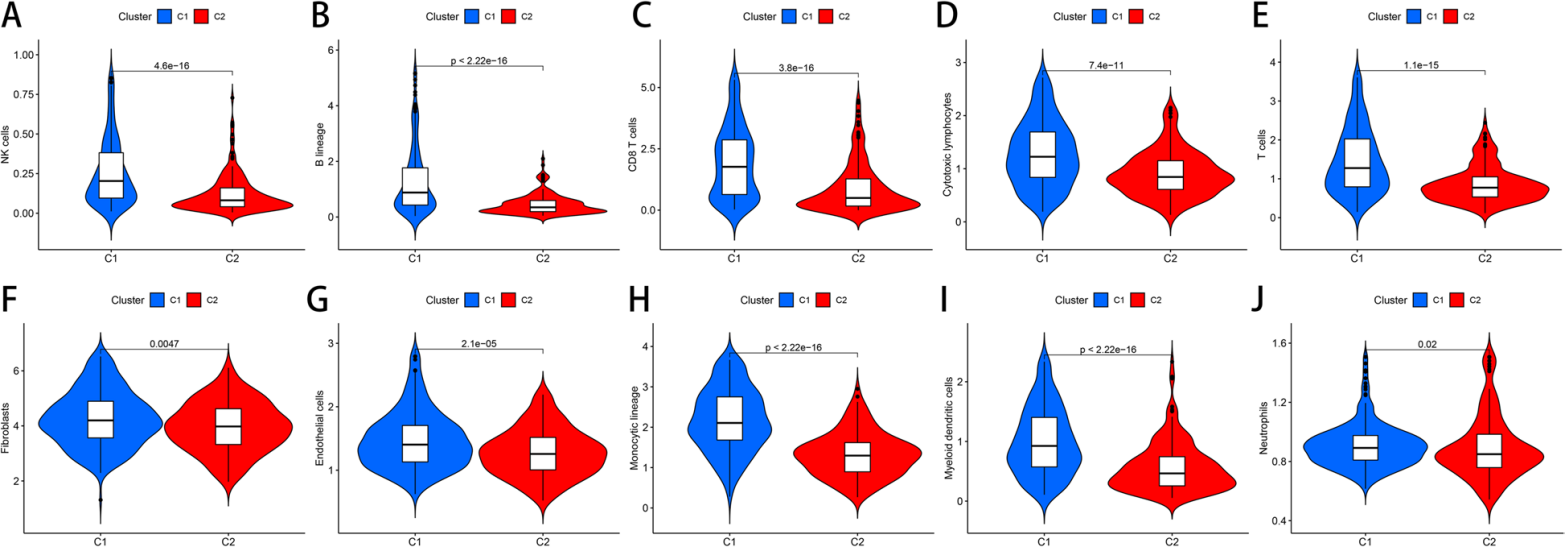
Violin plot differences in immune cell infiltration between two clustered samples

Under the MCPcounter algorithm, we found that in cluster1, the infiltration levels of T cells, CD T cells, Cytotoxic lymphocytes, B lineage, NK cells, Monocytic lineage, Myeloid dendritic cells, Neutrophils, Endothelial cells, and Fibroblasts were significantly increased (Fig. 3). This result helps us to explain that the prognosis of cluster1 patients is considerably better than that of cluster2, which may be attributed to the different levels of immune cell infiltration.

### Construction of a prognostic model based on HRGs

**Fig. 4:**
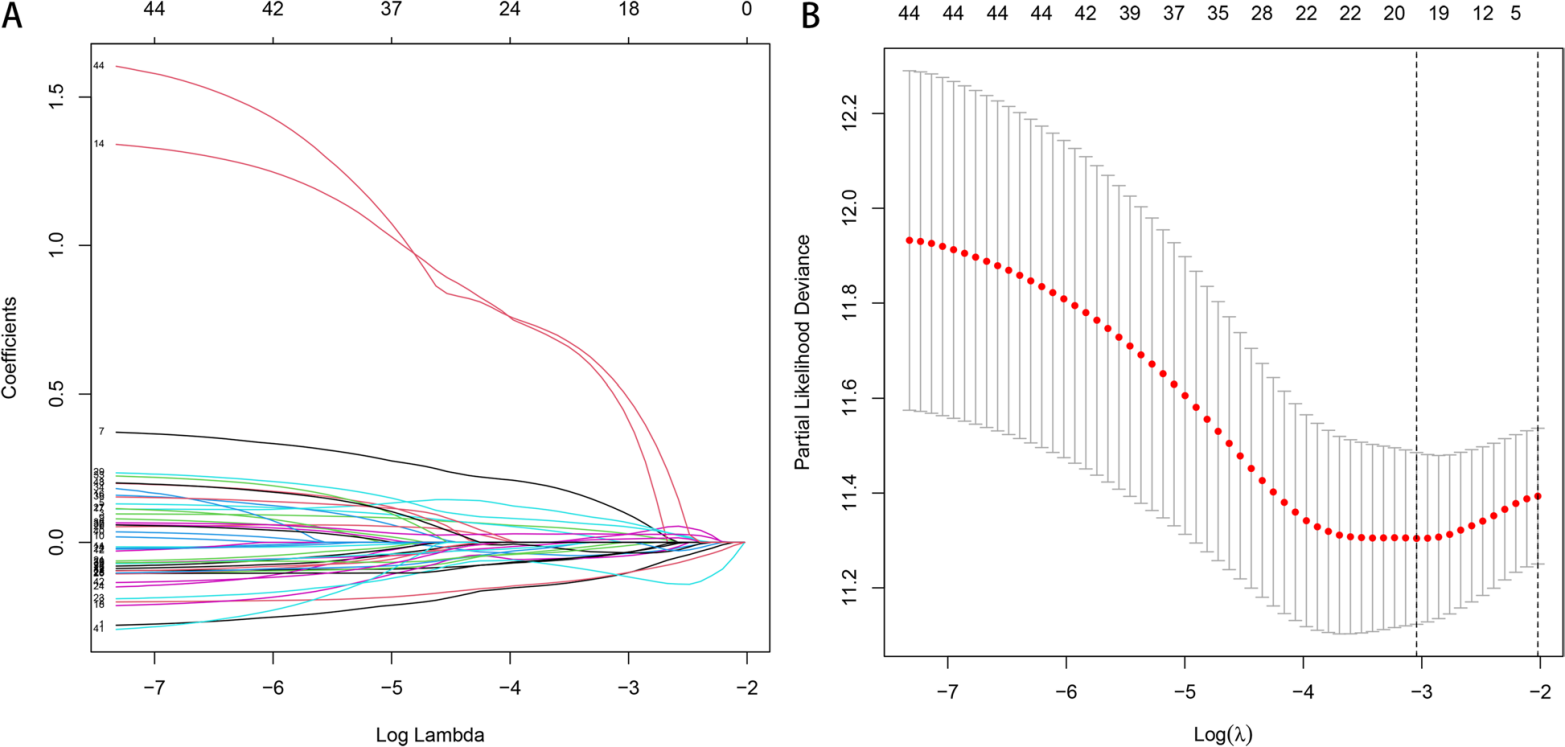
Construction of the prognostic model. **A** Ten-fold cross-validations for screening of the optimal parameter (lambda). **B** The optimal lambda determines LASSO coefficient profiles

**Fig. 5:**
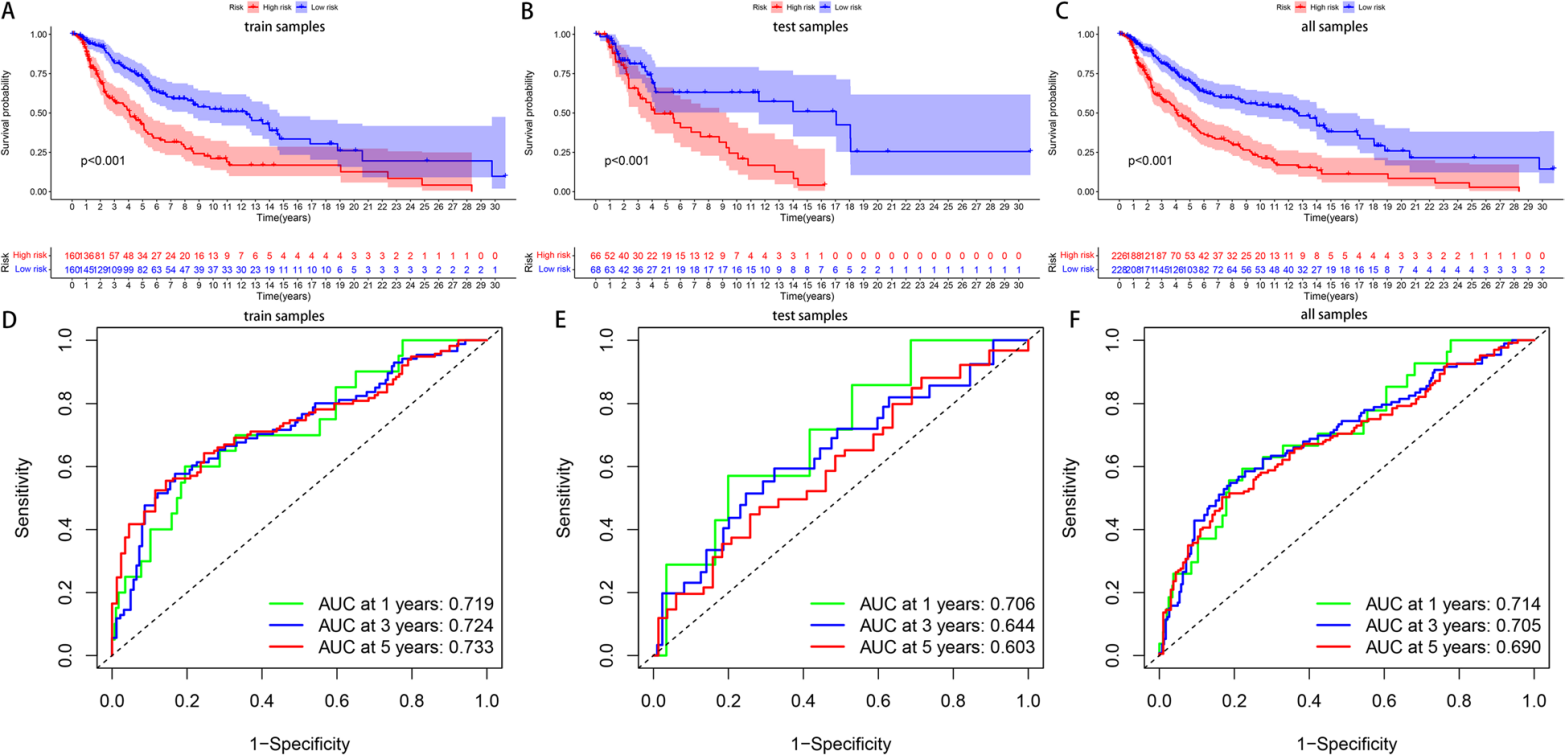
Survival curves of CM patients in high and low-risk groups. **A**-**C** The OS of the high-risk group was significantly shorter than that of the low-risk group. **D**-**F** The ROC and the areas under the curve verified the accuracy of prognostic signature in the groups

To screen out more important genes in melanoma, we identified 44 prognostic-related HRGs from 200 HRGs using univariate COX regression analysis for subsequent analysis (*p* < 0.05). And further used LASSO regression analysis to identify eight hub genes for constructing the prognostic model (Fig. 4A, B), including FBP1, NDRG1, GPI, IER3, B4GALNT2, BGN, PKP1, and EDN2. We randomly divide all samples into train and test groups, which helps us determine the model’s reliability. We further analyzed the relationship between melanoma patient outcomes by dividing patients into high- or low-risk groups based on risk scores. The results showed that patients in the low-risk group showed better overall survival than those in the high-risk group (Fig. 5A-C). Time‐dependent ROC analysis was applied further to evaluate the prediction efficiency of the constructed prognostic model. The areas under the curve (AUC) of all samples 1, 3, and 5 years being 0.719, 0.724, and 0.733, respectively; train group 1, 3, and 5 years being 0.706, 0.644, and 0.603, respectively; test group 1, 3 and 5 years being 0.714, 0.705 and 0.690, respectively (Fig. 5D-F). In addition, we performed separate survival analyzes on melanoma metastases and non-metastases to explore the role of the risk score. The results showed that samples from the low-risk group had better survival performance regardless of whether the tumor metastasized (Supplementary Fig. 4A, B). The AUC of non-metastatic tumor samples 1, 3, and 5 years being 0.717, 0.696, and 0.689, respectively; metastatic tumor samples 1, 3, and 5 years being 0.836, 0.875, and 0.875, respectively (Supplementary Fig. 4C, D).

### GSEA

**Fig. 6:**
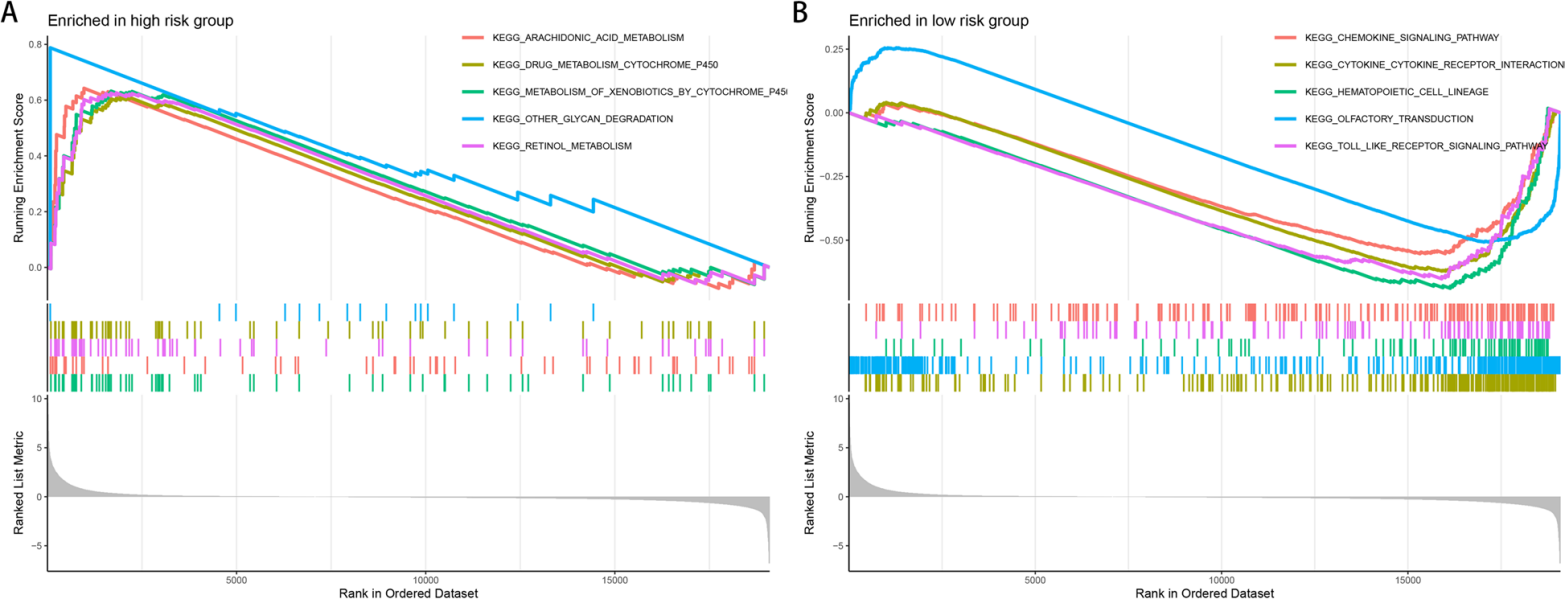
GSEA enrichment analysis plot. **A** GSEA enrichment pathway map of the high-risk group. **B** GSEA enrichment pathway map in the low-risk group

GSVA enrichment analysis showed that immune-related pathways, including TOLL-like receptor signaling and chemokine signaling, were highly expressed in low-risk samples compared with high-risk ones (Fig. 6A, B). The results of these gene functional enrichment analyses indicated that HRGs are closely related to immune regulation in melanoma patients, which may be the underlying mechanism for predicting the prognosis of melanoma patients.

### Relationship between HRGs and melanoma immune response

**Fig. 7:**
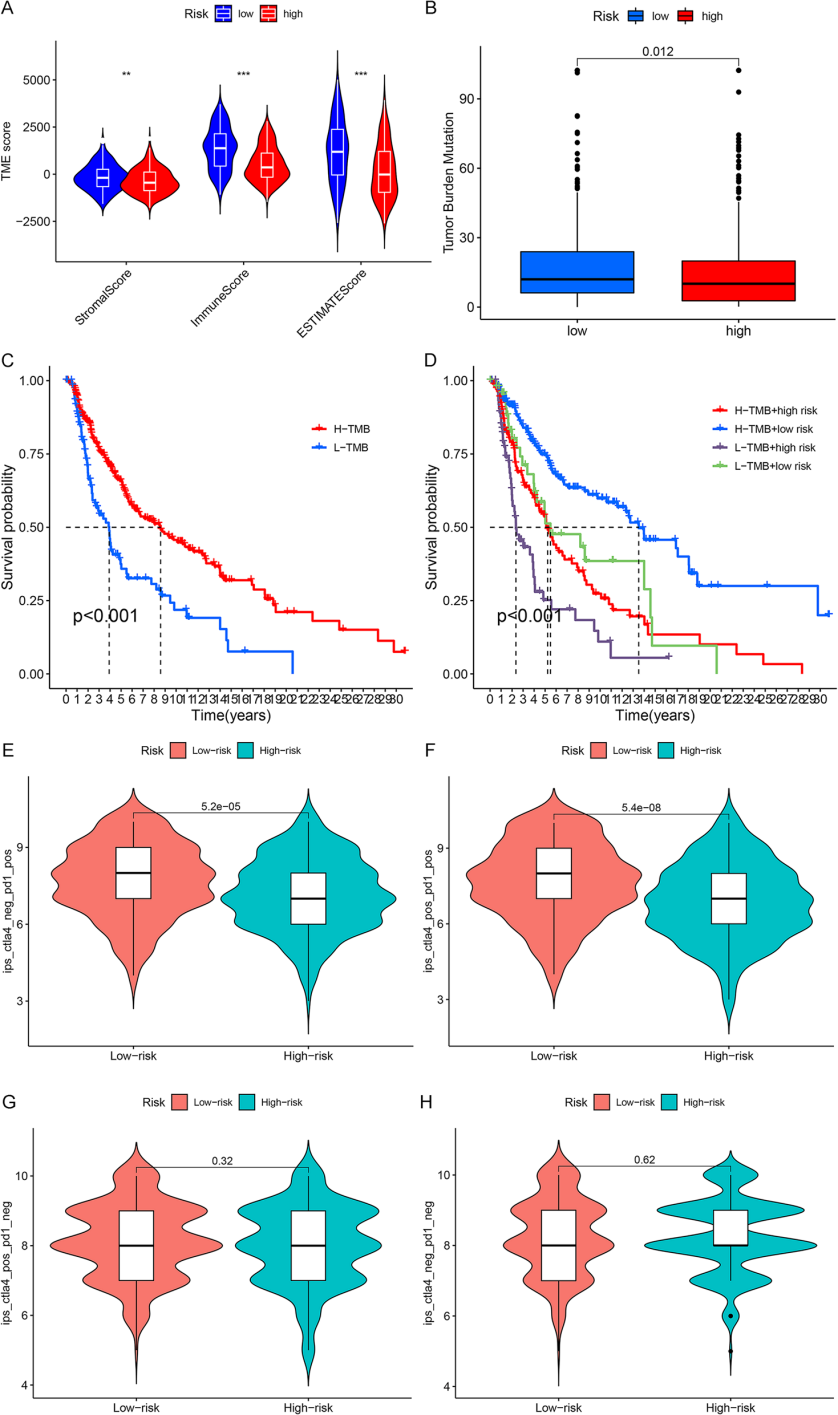
Relationship between HRGs and melanoma immune response. **A** Violin plot of correlation between risk score and TME index. **B** Boxplots of differences in TMB scores between high- and low-risk groups. **C** Survival curves of different TMB scores and CM patients. **D** Survival curves of different risk groups and different TMB scores and CM patients. **E** Distribution of risk scores in scores of CTLA4 negative and PD1 positive; **F** Distribution of risk scores in scores of CTLA4 positive and PD1 positive; **G** Distribution of risk scores in scores of CTLA4 positive and PD1 negative. **H** Distribution of risk scores in scores of CTLA4 negative and PD1 negative. *, *P* < 0.05; **, *P* < 0.01; ***, *P* < 0.001

To explore the possible role of HRGs in the TME, the link between HRGs expression and the TME index was analyzed [16, 17]. These immune indicators have enabled better prognosis assessment of tumor patients [18]. Our analysis showed that compared with the low-risk group, the high-risk group had significantly lower immune indicators, including stromal, immune, and ESTIMATE scores (Fig. 7A), which means that the low-risk group may have a better immune response. TMB is closely related to the prognosis of many tumor types after immunotherapy and can be used as a biomarker to predict the efficacy of immunotherapy [19]. In the low-risk group, TMB, a novel marker for immune checkpoint inhibitors (ICIs), was correspondingly elevated, reflecting that gene signatures may be promising markers for ICIs (Fig. 7B). Subsequent survival analysis also revealed that higher TMB scores were associated with better survival characteristics in melanoma (Fig. 7C). Combining risk scores, we obtained consistent results: samples with a higher TMB score with a low-risk score had the best prognosis, and samples with a lower TMB score with an increased risk score had the worst prognosis (Fig. 7D). Further, we validated the immune efficacy of the prognostic models via the TCIA database. The IPS values, which were calculated based on immunogenicity from the TCIA database, were analyzed in the risk model. The higher the IPS values, the better the effect on immunotherapy. The results showed that the IPS was obviously higher in the low-risk group, which indicated patients with lower risk would achieve a better response to immunotherapy (Fig. 7E, F). Specifically, the low- and high-risk groups had significant immunogenicity for PD1 immunotherapy (Fig. 7E: *p* = 5.2*10^–5^, Fig. 7F: *p* = 5.4*10^–8^), but not for CTLA4 immunotherapy (Fig. 7G: *p* = 0.32, Fig. 7H: *p* = 0.62). In addition, we also downloaded the GSE65904 dataset in the GEO database, and further performed survival and immune analysis to validate the results of TCGA (Supplementary Fig. 5). The results showed that the low-risk group also had better prognostic performance (*p* < 0.001), and the AUC of all samples 1, 3, and 5 years being 0.684, 0.683, and 0.678, respectively (Supplementary Fig. 5A, B). Since the TCIA database does not include data from the GEO database, we use the TIDE database to verify the relationship between risk scores and ICIs. The results showed that the high-risk group had higher TIDE scores (Supplementary Fig. 5C). Taken together, these data demonstrated that the low-risk group was more sensitive to potential surrogate markers of response to ICIs than the high-risk group.

## Discussion

More and more studies have confirmed that TME promotes cancer progression in many aspects, especially treatment resistance, which is very important in evaluating the efficacy of ICIs [20]. Hypoxic TME is prevalent due to increased oxygen consumption and abnormal proliferation in solid tumors. Hypoxia induces altered gene expression and subsequent proteomic changes that significantly affect various cellular and physiological functions, ultimately limiting patient outcomes [21]. Therefore, studying the role of HRGs in CM, especially the role of ICIs, can help us choose an appropriate treatment in the face of different CM patients.

In this study, we first used NMF clustering to extract the biological correlation coefficients of the data within the gene expression matrix and capture the internal structural features of the data, thereby dividing the CM samples into two groups. Subsequently, we performed survival analysis and differences in immune cell infiltration between the two groups of samples. In the better-surviving cluster1 samples, we found higher levels of immune cell infiltration, including T cells, CD8 T cells, Cytotoxic lymphocytes, B lineage, NK cells, Monocytic lineage, Myeloid dendritic cells, Neutrophils, Endothelial cells, and Fibroblasts. These immune cells kill tumor cells and can increase the efficacy of ICIs [22]. These results indicate that the expression level of HRGs is related to the prognosis of CM patients and may affect the level of immune cell infiltration in tumor tissue.

After identifying the possible roles of HRGs in CM, we need to further screen for gene signatures that may serve as prognostic markers. Through univariate COX regression analysis and LASSO regression analysis, we finally screened out 8 HRGs and constructed a prognostic model, including FBP1, NDRG1, GPI, IER3, B4GALNT2, BGN, PKP1, and EDN2. Indeed, common HRGs, FBP1, GPI, BGN, PKP1, and NDRG1, have been reported as risk factors in CM [23–27] and are associated with immune cell infiltration in the TME. IER3 is a potential prognostic factor in patients with hepatocellular carcinoma and acute myeloid leukemia [28, 29]. B4GALNT2 is down-regulated in colorectal and gastric cancers and elevated in breast cancer [30], and in CM, we report for the first time that it may act as a risk factor influencing patient prognosis. Likewise, EDN2 was revealed as a novel prognostic marker in various cancers, including prostate cancer [31] and breast cancer [32]. Here, for the first time, we identified a gene signature composed of eight genes, FBP1, NDRG1, GPI, IER3, B4GALNT2, BGN, PKP1, and EDN2 in CM, and the risk score was composed of coefficients, more than using a single biomarker alone. Each gene's expression status significantly improves diagnostic results' reliability and accuracy. Through validation in the train and test groups, we determined that patients in the low-risk score group imply a better prognosis.

In terms of the efficacy of ICIs, we first determined that in the high-risk group samples, immune indicators were significantly reduced, including stromal, immune, and ESTIMATE scores, which reflected that the low-risk group samples might have a better immune response. Second, TMB has been recognized as a biomarker for predicting the efficacy of immunotherapy [19]. Our results also showed a corresponding increase in TMB in the low-risk group, reflecting that gene signatures may be promising markers for ICIs. Subsequent survival analyses also showed that higher TMB scores were associated with better survival characteristics in melanoma. Therefore, we established a new 8-gene signature, a promising tool for predicting CM prognosis and immune efficacy. Melanoma's development and progression have been linked to various components of the immune system [33, 34]. In the context of tumor immunity, tumor cells are seen as antigens, and immune cells and leukocytes serve as the immune defense by infiltrating the tumor tissue through chemotaxis [33]. While there are several new immunotherapies available for melanoma treatment, such as PD-1, PD-L1, and CTLA-4 inhibitors, these approaches are only effective for a small fraction of patients [35, 36]. Additionally, most patients show limited or no response to treatment, especially after the distant metastasis of melanoma [37, 38]. Our study has identified novel genetic signatures that can predict the potential efficacy of ICIs in patients with CM. Samples with low risk scores have shown significant immunogenicity for PD1 immunotherapy, indicating their potential usefulness in personalized therapy for CM patients. Additionally, some of the genes that make up the gene signature have been associated with CM-related immune infiltration. Fructose-bisphosphatase 1 (FBP1), traditionally known as the rate-limiting enzyme in gluconeogenesis, is highly expressed in CM and is associated with poor prognosis. When combined with other genes, FBP1 can be used to predict the immune microenvironment of CM [23, 39]. N-Myc Downstream Regulated 1 (NDRG1), a common tumor risk factor, is a powerful immune-related gene in CM and is associated with the ferroptosis pathway [24, 40]. Plakophilin 1 (PKP1) is overexpressed in metastatic melanoma and is a factor of poor prognosis in patients with CM, suggesting a link between PKP1 gene expression and immune efficacy [27]. Our study represents a groundbreaking contribution as it unveils the previously unexplored association between GPI, IER3, B4GALNT2, BGN, and EDN2 with the immune microenvironment in CM. Moreover, we have successfully developed a risk score, which enhances the precision of predicting surrogate markers of response to ICIs, including TMB, IPS values, and TIDE scores. However, these results still need to be supplemented by further basic experiments.

## Conclusion

In conclusion, we reported the association of HRGs with patient prognosis in melanoma and screened for novel gene signatures, including FBP1, NDRG1, GPI, IER3, B4GALNT2, BGN, PKP1, and EDN2. Our results suggest that the gene signature composed of these eight genes could also provide novel insights into immunological biomarkers and the underlying mechanism of CM.

## Supplementary Information

**Additional file 1: Supplementary Table 1.** Clinical characteristics of the GSE65904 dataset.

**Additional file 2: Supplementary Fig. 1.** NMF rank analysis. **Supplementary Fig. 2.** The heatmap of NMF. **Supplementary Fig. 3.** The expression of HRGs. **Supplementary Fig. 4.** Survival analysis. **Supplementary Fig. 5.** Survival and Immunotherapy Efficacy Analysis.

## Data Availability

The data used in this study is freely available in the TCGA online database (https://portal.gdc.cancer.gov/repository?facetTab=files&filters=%7B%22op%22%3A%22and%22%2C%22content%22%3A%5B%7B%22op%22%3A%22in%22%2C%22content%22%3A%7B%22field%22%3A%22cases.disease_type%22%2C%22value%22%3A%5B%22adenomas%20and%20adenocarcinomas%22%5D%7D%7D%2C%7B%22op%22%3A%22in%22%2C%22content%22%3A%7B%22field%22%3A%22cases.primary_site%22%2C%22value%22%3A%5B%22stomach%22%5D%7D%7D%2C%7B%22op%22%3A%22in%22%2C%22content%22%3A%7B%22field%22%3A%22cases.project.program.name%22%2C%22value%22%3A%5B%22TCGA%22%5D%7D%7D%2C%7B%22op%22%3A%22in%22%2C%22content%22%3A%7B%22field%22%3A%22cases.project.project_id%22%2C%22value%22%3A%5B%22TCGA-STAD%22%5D%7D%7D%2C%7B%22op%22%3A%22in%22%2C%22content%22%3A%7B%22field%22%3A%22files.analysis.workflow_type%22%2C%22value%22%3A%5B%22STAR%20-%20Counts%22%5D%7D%7D%2C%7B%22op%22%3A%22in%22%2C%22content%22%3A%7B%22field%22%3A%22files.data_category%22%2C%22value%22%3A%5B%22transcriptome%20profiling%22%5D%7D%7D%2C%7B%22op%22%3A%22in%22%2C%22content%22%3A%7B%22field%22%3A%22files.data_type%22%2C%22value%22%3A%5B%22Gene%20Expression%20Quantification%22%5D%7D%7D%2C%7B%22op%22%3A%22in%22%2C%22content%22%3A%7B%22field%22%3A%22files.experimental_strategy%22%2C%22value%22%3A%5B%22RNA-Seq%22%5D%7D%7D%5D%7D) and GSE65904 from the GEO database (https://www.ncbi.nlm.nih.gov/geo/). Our analyses, protocols, raw figures, or other information related to this study could be requested from the corresponding author(s) upon reasonable request.

## Abbreviations

LASSO: Least Absolute Shrinkage and Selection Operator
ROC: Receiver operating characteristic curve
AUC: Area under the curve
DEGs: Differentially expressed genes
TME: Tumor microenvironment
ICI: Immune checkpoint inhibitor
