# Supplementary material for: Explore the impact of hypoxia-related genes (HRGs) in Cutaneous melanoma
Source: BMC Med Genomics. 2023 Jul 8;16:160. doi: 10.1186/s12920-023-01587-8 (PMC10329328; doi:10.1186/s12920-023-01587-8)
Supplement: Supplementary file 1 — Additional file 1: Supplementary Table 1. Clinical characteristics of the GSE65904 dataset. [file 12920_2023_1587_MOESM1_ESM.docx]

Supplementary Table 1: Clinical characteristics of the GSE65904 dataset.

| Patient characteristics | Whole cohort (N=214) |
| --- | --- |
| Gender, n (%) |  |
| Male | 124 (58) |
| Female | 89 (42) |
| NA | 1 (0.5) |
| Age, n (%) |  |
| <60 | 80 (37) |
| ≥60 | 130 (61) |
| NA | 4 (2) |
| Tumor type, n (%) |  |
| Primary | 16 (7) |
| Metastasis | 188 (88) |
| NA | 10 (5) |
| Metastasis type, n (%) |  |
| Local | 11 (5) |
| In-transit | 15 (7) |
| Regional | 139 (65) |
| General | 23 (11) |
| NA | 26 (12) |
