# Supplementary material for: Explore the impact of hypoxia-related genes (HRGs) in Cutaneous melanoma
Source: BMC Med Genomics. 2023 Jul 8;16:160. doi: 10.1186/s12920-023-01587-8 (PMC10329328; doi:10.1186/s12920-023-01587-8)
Supplement: Supplementary file 2 — Additional file 2: Supplementary Fig. 1. NMF rank analysis. Supplementary Fig. 2. The heatmap of NMF. Supplementary Fig. 3. The expression of HRGs. Supplementary Fig. 4. Survival analysis. Supplementary Fig. 5. Survival and Immunotherapy Efficacy Analysis. [file 12920_2023_1587_MOESM2_ESM.pdf]

Supplementary figures

Supplementary fig. 1: NMF rank analysis.

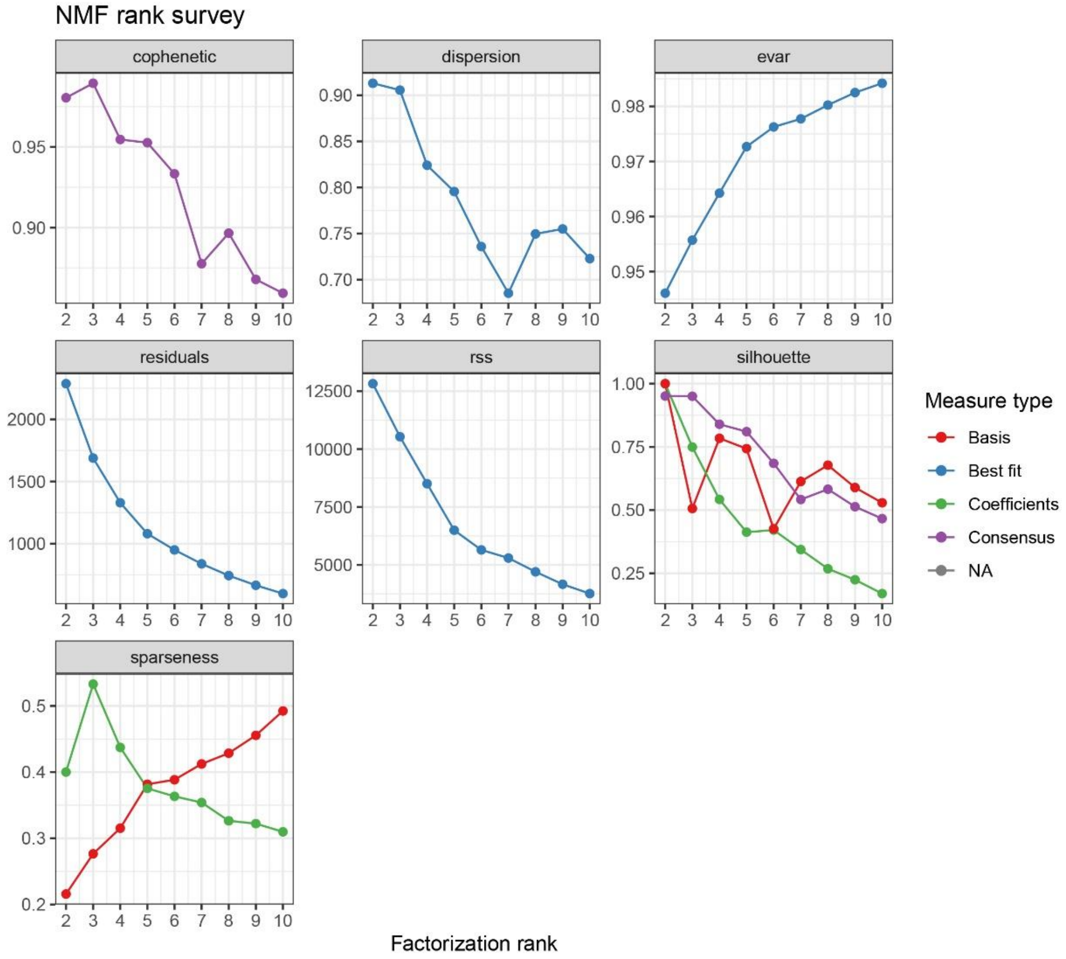

Supplementary fig. 1: The relationship between cophenetic, dispersion and silhouette coefficients with respect to number of clusters.

Supplementary fig. 2: The heatmap of NMF.

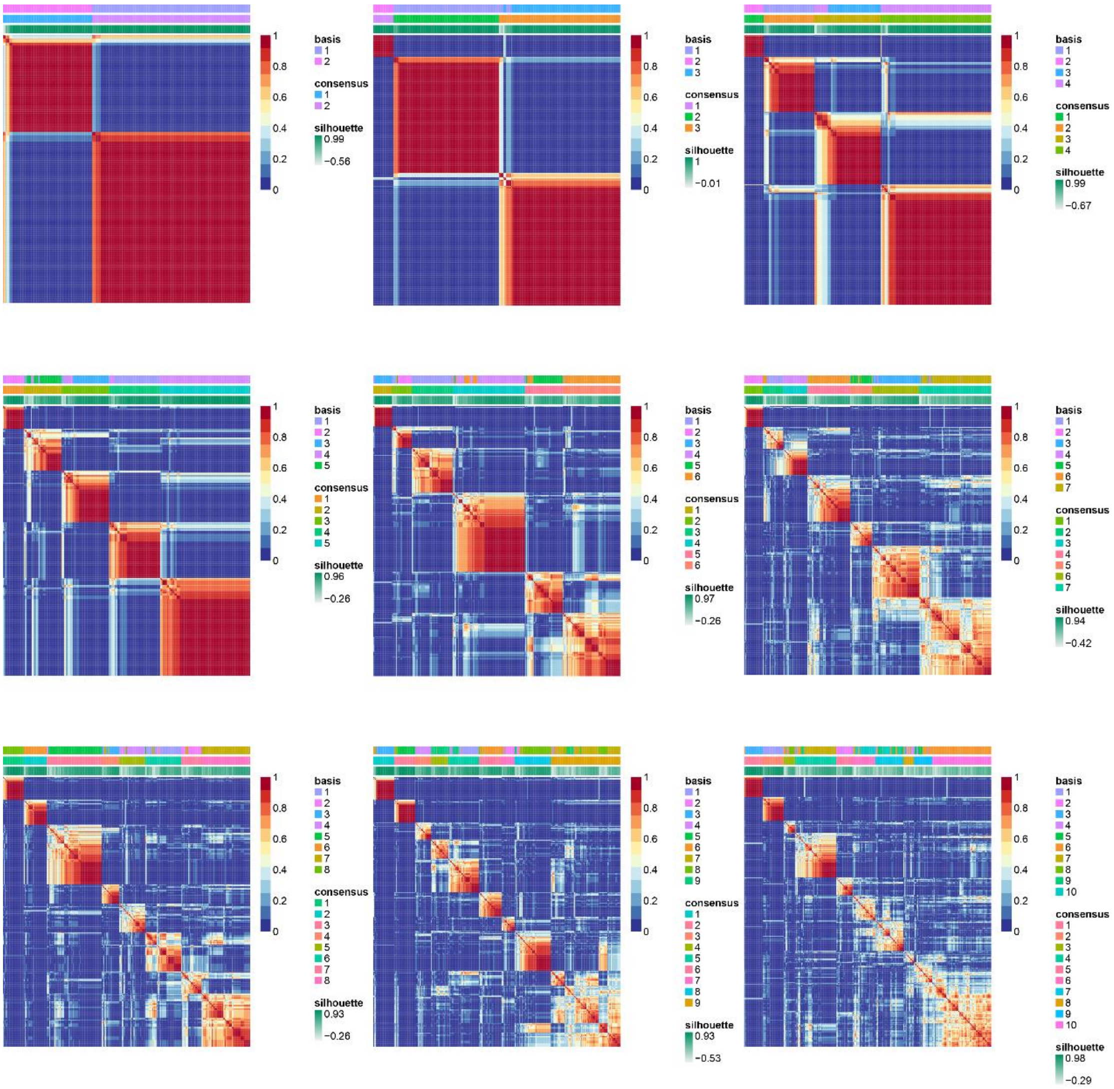

Supplementary fig. 2: Heatmap representation of NMF clustering.

Supplementary fig. 3: The expression of HRGs.

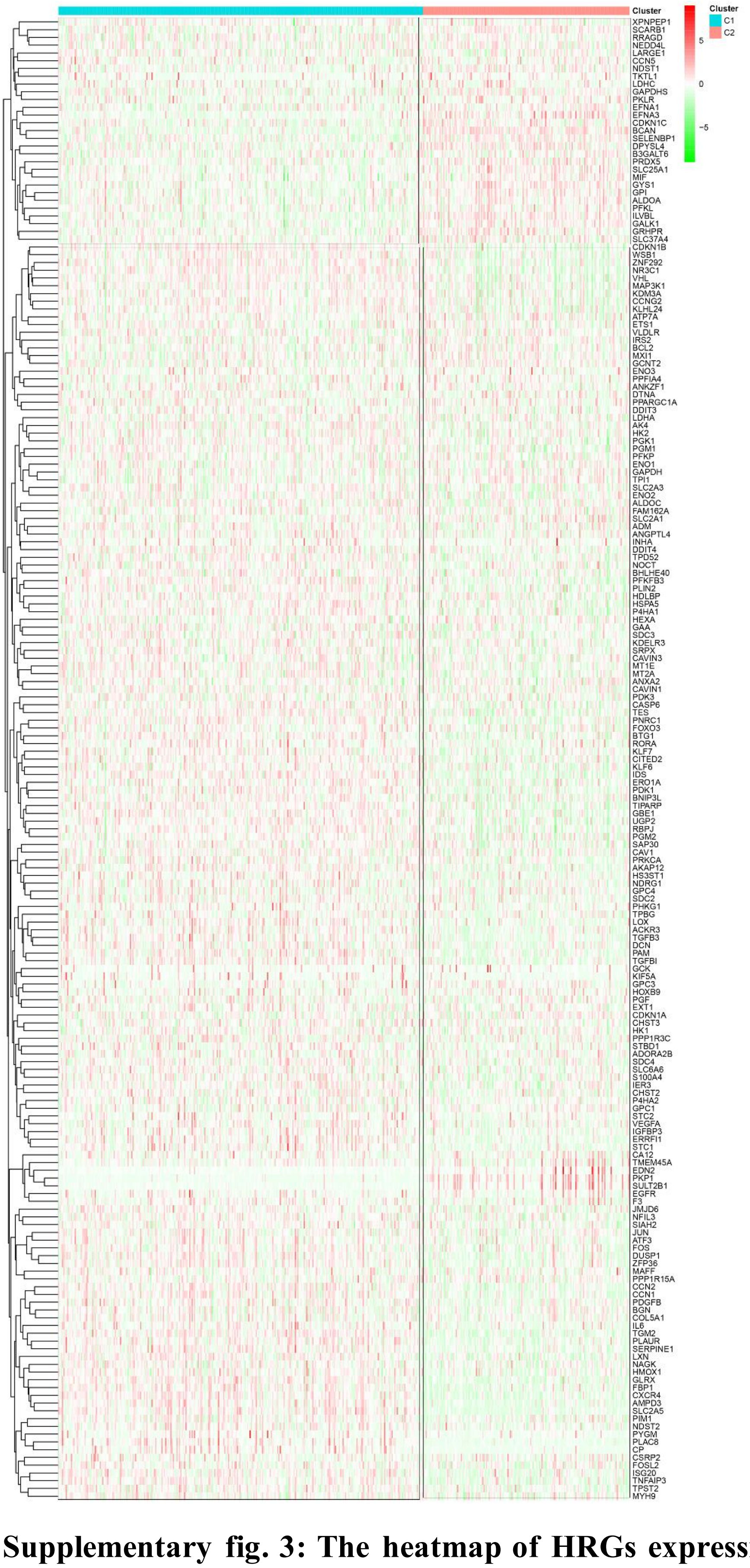

Supplementary fig. 3: The heatmap of HRGs expression in different

clusters.

Supplementary fig. 4: Survival analysis.

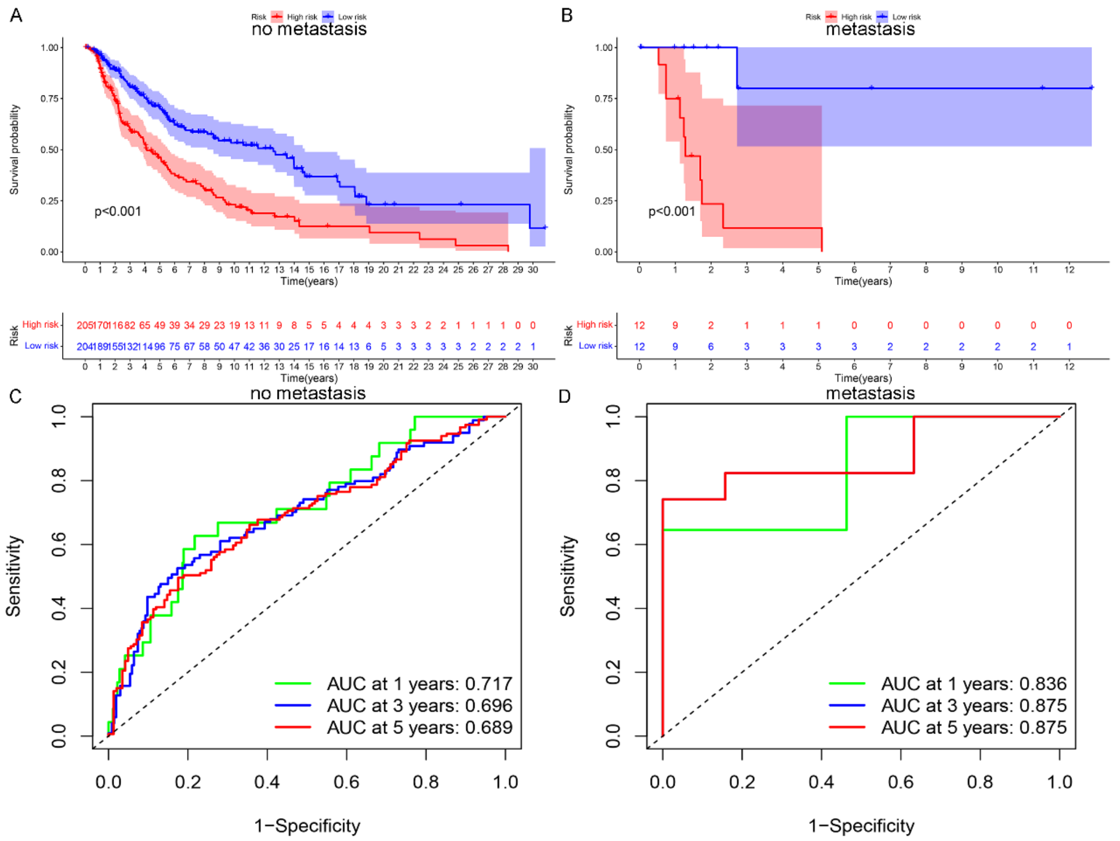

Supplementary fig. 4: Survival curves and ROC curves of tumor metastasis samples and non-metastatic samples. (A&B) Prognostic analysis and ROC curves of high-risk and low-risk groups in non-metastatic samples. (C&D) Prognostic analysis and ROC curves of high-risk and low-risk groups in tumor metastasis samples.

Supplementary fig. 5: Survival and Immunotherapy Efficacy Analysis.

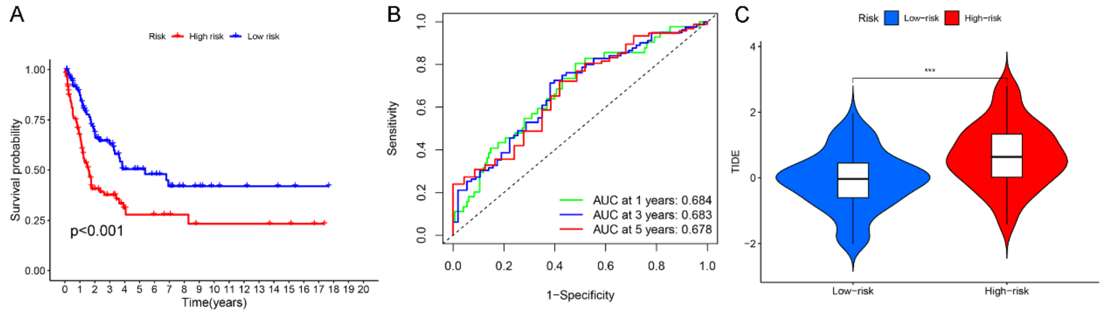

Supplementary fig. 5: Survival and Immunotherapy Efficacy Analysis. (A&B) In the GSE65904 data set, the survival curve and ROC curve of the risk score and the prognosis of tumor patients. (C) Comparison of TIDE scores for high-risk and low-risk samples.
